# Radiographic, antibacterial and bond-strength effects of radiopaque caries tagging

**DOI:** 10.1038/srep27319

**Published:** 2016-06-02

**Authors:** Aurore Umwali, Haitham Askar, Sebastian Paris, Falk Schwendicke

**Affiliations:** 1 Department of Operative and Preventive Dentistry, Charité – Universitätsmedizin Berlin, Aßmannshauser Str. 4-6, 14199 Berlin, Germany

## Abstract

Selectively excavated carious lesions remain radiographically detectable. Radiopaque tagging could resolve the resulting diagnostic uncertainty. We aimed to evaluate if tagging depends on lesions depths, is antibacterial, or affects dentin bond-strengths. Artificial lesions (depth-range: 152–682 μm, n = 34/group) were induced in human dentin samples, evaluated using wavelength-independent microradiography, treated with one of two tagging materials (70% SnCl_2_, 30% SnF_2_) and re-evaluated. To evaluate antimicrobial effects, 40 dentin samples were submitted to a *Lactobacillus rhamnosus* invasion-model. Infected *s*amples were treated with placebo, 0.2% chlorhexidine, SnCl_2,_ SnF_2_ (n = 10/group). Dentin was sampled and colony-forming units/mg determined. Micro-tensile bond-strengths of adhesive restorations (OptiBond FL, Filtek Z250) to tagged or untagged, sound and carious dentin were assessed (n = 12/group). Tagged surfaces were evaluated microscopically and via energy-dispersive X-ray-spectroscopy (EDS). Tagging effects of both materials decreased with increasing lesion depths (p < 0.001). Un-/chlorhexidine-treated dentin contained significantly more viable bacteria (median 7.3/3.7 × 10^5^ CFU/mg) than tagged dentin (no CFU detectable, p < 0.001). Tagging decreased bond strengths (p < 0.001) on sound (−22%/−33% for SnCl_2_/SnF_2_) and carious dentin (−50%/−54%). This might be due to widespread tin chloride or fluoride precipitation, as detected via microscopy and EDS. While radiopaque tagging seems beneficial, an optimized application protocol needs to be developed prior clinical use.

Treatment of deep carious lesions usually involves removal of carious dentin followed by restoration of the cavity. Given the risks of removing all carious (demineralized, softened) dentin in proximity to the pulp, less invasive removal strategies like stepwise or selective (incomplete, partial) excavation have been shown advantageous for treating deep lesions, as they reduce the risks of pulpal exposure and complications^1^,^2^.

Despite growing evidence supporting such excavation strategies, most dentists do not perform stepwise or, even less so, selective carious tissue removal^3^,^4^,^5^. One often mentioned reason for this reluctance is that residual lesions sealed beneath restorations remain radiographically detectable as translucency. This translucency cannot be distinguished from overseen or recurrent, progressive lesions^6^, and might be misinterpreted by other dentists, who are not familiar with selective excavation and the specific history of the tooth. Consequently, such sub-restoration translucencies could be seen as indication for a faulty restoration and trigger replacement, which would thwart the originally minimal-invasive treatment concept.

Cavity pretreatment with radiographic tagging materials, for example using highly concentrated stannous chloride or fluoride solutions, has been suggested to overcome this problem. Such tagging masks intentionally left carious dentin, thus preventing unnecessary re-treatment, and allows to discriminate arrested from progressing lesions^6^. So far, it has not been assessed if tagging effects of residual lesions depend on lesion depth or mineral loss. It could be speculated that full penetration and camouflaging might be achieved in shallow, but not deeper lesions, which would mean that this technique is not suitable for all indications, limiting its clinical applicability. Tagging was further speculated to be beneficial by exerting antibacterial effects, acting as a cavity disinfection. Such disinfection could decrease the bacterial load remaining beneath restorations, and could increase the acceptability of selective excavation. Last, tagging using highly concentrated tin compounds could affect dentin bond strengths. Lowly concentrated stannous chloride (applied to protect dentin from acid erosion) was found to increase bond strength of self-etch adhesives, while bond strengths of etch-and-rinse adhesives were decreased^7^. Bond strength effects of tagging materials might further differ depending on sound or carious dentin being the substrate^8^.

Therefore, the present study aimed to assess if tagging effects differ in differently deep lesion, and if tagging has antibacterial effects or affects dentin bond strength *in vitro.* We hypothesized that the relative tagging effect would significantly decrease in deeper lesions, that tagging would significant decrease dentin bacterial numbers, and that tagging would significantly reduce dentin bond strength.

## Methods

### Study design

Two tagging materials, 70% SnCl_2_ and 30% SnF_2_ dissolved in distilled water, were used for all experiments. Tagging of differently deep artificial carious lesions was evaluated using transversal wavelength-independent microradiography (T-WIM)^9^. Antibacterial effects of tagging materials were tested using artificial, bacterially-invaded residual lesions. Effects of tagging on bond strengths of adhesive restorations to sound and carious dentin was evaluated via micro-tensile bond strength (μTBS) measurements.

### Tagging of differently deep lesions

80 sound extracted human permanent molars were obtained under an ethics-approved protocol (Ethics Committee of the Charité, Berlin, EA4/102/14). Handling of teeth was carried out in accordance with this approval. Teeth were collected under informed consent. 170 dentin samples (3 × 2 × 1 mm) were cut (Band Saw EXAKT 300 CL, EXAKT Advanced Technologies, Norderstedt, Germany) to expose the coronal dentin, which was then polished up to 4000 grit (SiC paper, Struers, Willich, Germany). We carefully checked for removal of all enamel. Afterwards, samples were split sagittally and prepared for T-WIM^10^. Half of the coronal surface of each sample was covered with nail-varnish (Riva-De-Loop, Rossmann, Burgwedel, Germany). Artificial lesions were created in unprotected areas by samples storage in 5 l demineralizing solution^11^ at pH 5.3 and 37 °C under agitation for 2, 4, 6, 8 and 10 w (n = 34/group). Baseline integrated mineral loss (∆Z) and lesion depths (LD) of induced deep lesions were assessed using T-WIM. Demineralized areas were gently air-dried for 5 s, and SnCl_2_ or SnF_2_ (Sigma Aldrich, St. Louis, USA) applied twice for 15 s using brushing movements, followed by removal of excess material with micro-brushes (use of blotting paper etc. for removal is unlikely in a clinical setting).

The resulting tagging effect (∆∆Z) was evaluated using T-WIM as described using 35 mm films (B/W positive, Fujifilm, Tokyo, Japan)^10^. Microradiographs were microscopically assessed (Axioplan 60318, Zeiss, Oberkochen, Germany), digitalized (CFW1312M, Scion, Frederick, USA) and evaluated (TMR 2000 2.0.27.2, UMCG, Groningen, Netherlands). ∆∆Z was calculated relative to baseline values (∆∆Z [%] = −[∆Z_tagging_ − ∆Z_baseline_] × ∆Z^−1^), with positive values indicating reduced translucency.

### Antibacterial effects

To evaluate antimicrobial effects, 40 dentin samples (3 × 3 × 2 mm) were prepared from caries-free human molars as described. Samples were embedded (Technovit), with the occlusal surface being uncovered, pre-demineralized using 0.5 M EDTA for 24 h, followed by acetic acid demineralization using identical conditions as described above for 14 days. Samples were sterilized (121 °C, 2.4 bar) and submitted to a constant-culture bacterial invasion-model employing *Lactobacillus rhamnosus (*ATCC 12116). Briefly, 15 ml culture medium (deMan Rogosa Sharpe [MRS] plus 5% sucrose) was supplied together with overnight cultures of *L. rhamnosus* (approx. 10^8^ CFU/ml) for 24 h at 37 °C. Fluids were then removed to let the developed biofilms rest for 2 h. Afterwards, MRS-S was slowly rinsed over the samples (1 ml/min) for 15 min using peristaltic pumps. Medium was then removed once more, and biofilms rested again for 2 h. This was repeated 6× daily. Overnight cultures of *L. rhamnosus* were provided once daily. After 2 w, samples were removed, cleaned from overlying biofilm, and divided into 4 groups: Placebo treatment (20 μl phosphate buffered saline applied twice for 15 s), 0.2% chlorhexidine (positive control, Sunstar, Schönau, Germany, applied twice for 15 s as described), SnCl_2_ and SnF_2_, also applied as described (n = 10/group). Afterwards, dentin was sampled using sterile rose-head burs (Komet, Lemgo, Germany), which had been weighed upfront. All softened dentin was removed, and burs with dentin weighed once more. Dentin was then transferred to 0.9% sodium chloride, serially diluted from 10^−1^–10^−5^, and plated onto MRS agar for enumeration of colony-forming units (CFU) per mg dentin after 48 h.

### Bond-strength effects

Thirty-two extracted human molars were completely embedded in methacrylate resin (Technovit 4071, Heraeus Kulzer, Hanau, Germany). Occlusal surfaces were exposed and polished as described. Note that the resulting surfaces are relatively smooth, which could result in different bonding strengths when compared to other studies regardless of group allocation. Comparison between studies should thus be made with caution.

On the polished surface of 24 teeth, artificial carious lesions were induced as described using acetic acid solution (pH 5.3, 37 °C) for 40 days. The remaining samples were stored in distilled water. Afterwards, 4 sound and 4 artificially carious teeth were allocated to control (no radiopaque tagging), SnCl_2_ and SnF_2_. In all groups, surfaces were etched for 20 s using 37% phosphoric acid (ANA, Nordiska, Ängelholm, Sweden) for 15 s. For both test groups, radiopaque materials were applied twice (2 × 20 μl) for a total of 30 s. Restorations were placed using OptiBond FL (Kerr, Bioggio, Switzerland) and the resin composite Filtek Z250 (3M Espe, St. Paul, USA) according to manufacturer’s instructions. Composite was incrementally built up to a thickness of 6 mm. Increments were light-cured using an LED curing light (Valo, Ultradent, Salt Lake City, USA) with an intensity of 1400 mW/cm^2^, for 40 s. After 24 h storage in distilled water at 37 °C, samples were cut with a low-speed saw (Isomet 1000; Buehler, Lake Bluff, USA) under continuous water cooling to obtain sticks with a rectangular cross-section area of approximately 1 mm^2^, the width and breadth of the sticks were determined with a digital caliper. From each tooth three sticks were randomly selected form the most central dentin area, leading to n = 12 per group.

For micro-tensile testing, sticks were attached to the test assembly using modeling wax. No pretesting failures occurred. The tensile load was applied at a crosshead speed of 0.5 mm/min until the fracture occurred (Zwick, Ulm, Germany). Maximum force recorded before fracture was used to calculate the micro-tensile bond strength (μTBS = maximum load at failure/cross-section area). After each test, stick fragments were carefully removed from the test assembly and examined under stereomicroscope (Stemi, Zeiss, Oberhausen, Germany) at 45× magnification to detect the failure mode, which was classified as (1) cohesive failure in dentin, (2) adhesive failure between adhesive system and dentin, (3) adhesive failure between adhesive system and composite, (4) mixed adhesive failure (failure modes 2 and 3), (5) cohesive failure in composite.

### Scanning electron microscopy (SEM) and Energy dispersive x-ray spectroscopy (EDS)

Samples were air-dried and gold sputtered for SEM or carbon coated for EDS. SEM and EDS element mappings were performed in a CamScan MaXim scanning electron microscope (CamScan, Cambridge, UK), equipped with a Bruker XFlash 6 30 detector and ESPRIT 2.0 software (Bruker Nano, Berlin, Germany) at an acceleration voltage of 15 kV. The acquisition time for each element mapping was 300 sec.

### Statistical evaluation

Statistical analysis was performed using SPSS 20 (IBM, Armonk, USA). Normal distribution was assessed using Shapiro-Wilk-test. Effects of tagging on ∆Z were analyzed using ordinary least square regression analysis. Groupwise comparison were performed using two-sided Mann-Whitney-U-test. Level of significance was set at α = 0.05.

## Results

### Tagging of differently deep lesions

**Figure 1:**
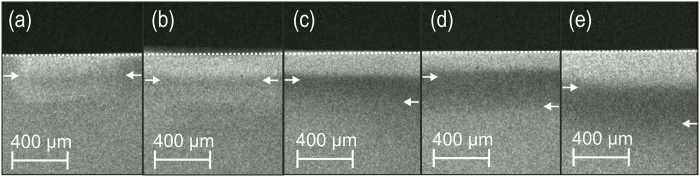
Transversal wave-length independent microradiographic images of lesions with different lesion depths (from (**a–e**)) after radiopaque tagging with SnCl_2_. Arrows indicate the lesion depth (right arrow in each panel) and the lesion depth (left arrow).The relative tagging effect decreases with increasing lesion depth.

**Figure 2:**
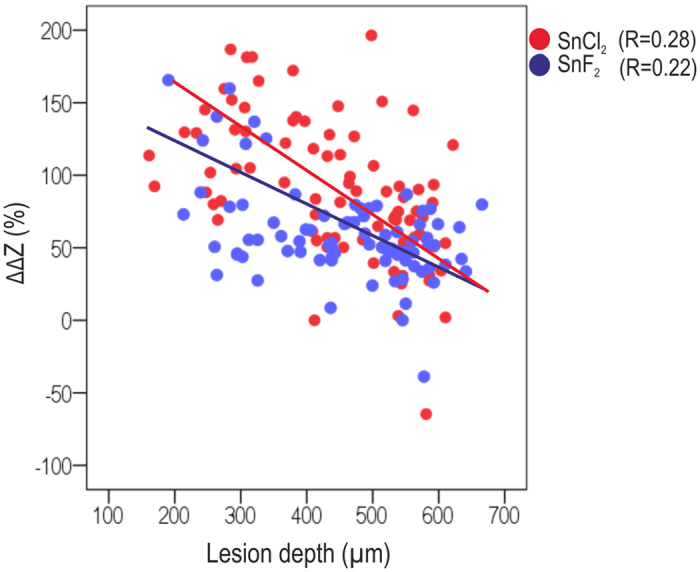
Radiopaque tagging of differently deep lesions. Mineral loss (ΔZ) was measured at baseline. After radiopaque tagging using stannous chloride (red) and fluoride (blue), relative differences (ΔΔZ%) were determined. Significant associations with between lesion depth and tagging effects were identified (R = linear model fit). For SnF_2_, an inverse function was also found to describe the association (R^2^ = 0.27).

Using T-WIM, we found the induced artificial lesions to have mean mineral losses [ΔZ] between 3560 (SD: 1050) after 2 weeks of demineralization to 5005 (1470) vol%×μm after 10 weeks, with lesion depths between 152 μm and 682 μm (Fig. 1). Relative tagging effects were significantly higher in SnCl_2_ than SnF2 (p < 0.05), but decreased with increasing lesion depths for both tagging materials (Fig. 2).

### Antibacterial effects

**Figure 3:**
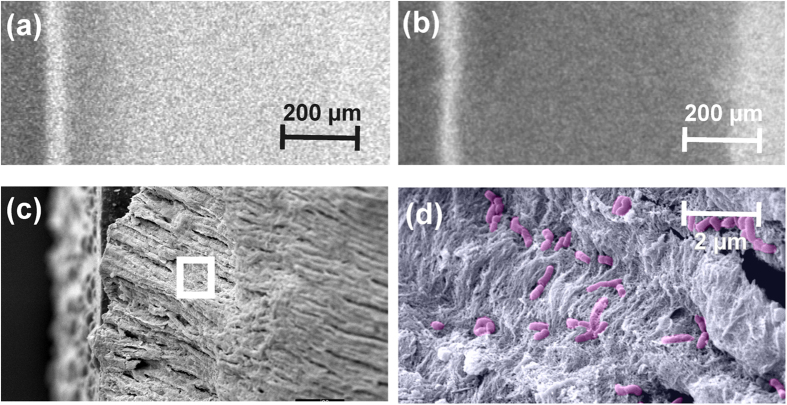
Induction of artificial residual lesions. Lesions were first induced via EDTA and acetic acid, leading to clearly detectable mineral loss as per microradiography (**a**). Samples were then submitted to a constant-culture biofilm model employing *Lactobacillus rhamnosus*, significantly increasing mineral loss and lesion depth (**b**). The resulting dentin lesions contained a median of 7.3 (0.5/10.4) × 10^5^ CFU/mg. Bacteria could also be detected via scanning electron microscopy ((**c**) overview, (**d**) insert from (**c**), bacteria colored in pink).

**Figure 4:**
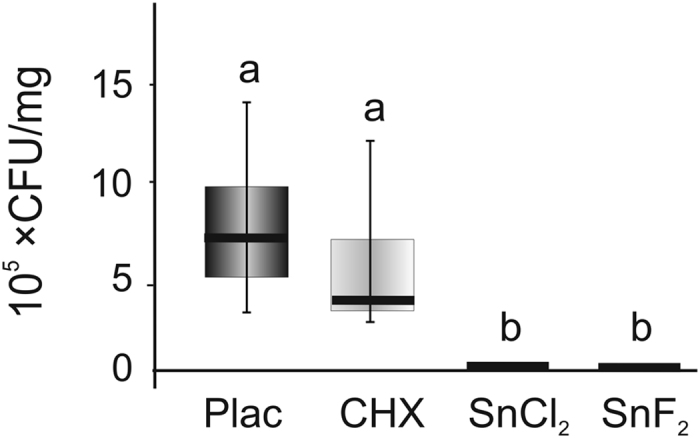
Bacteria load (10^5^× CFU/mg) in differently treated lesions. Lesions were treated using placebo (plac) treatment or 0.2% chlorhexidine (CHX), stannous chloride or stannous fluoride (n = 10/group). Different letters indicate statistical significance between groups (p < 0.001). Box and line: 25th/75th percentiles and median, whiskers: minimum and maximum.

Induced lesions (Fig. 3) harbored a median (25^th^/75^th^ percentiles) of 7.3 (5.0/10.4) × 10^5^ CFU/mg. Treatment with CHX did not significantly decrease the number of viable bacteria (Fig. 4), while no bacteria could be detected at 10^−1^ dilution after SnCl_2_- and SnF_2_-treatment (p < 0.001).

### Bond strengths effects

**Figure 5:**
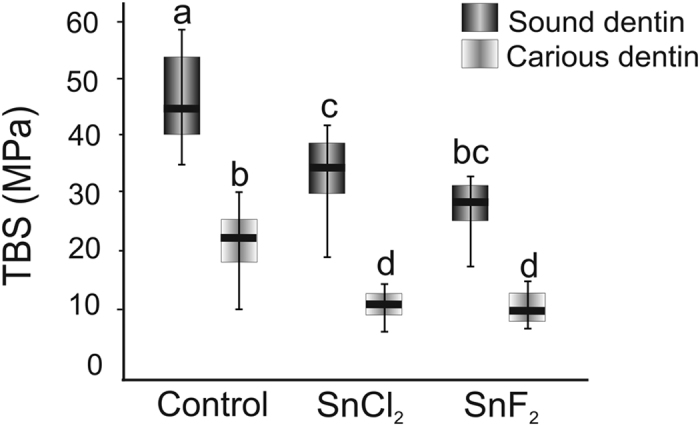
Micro-tensile bond strengths (TBS, in MPa) in different groups. TBS was assessed on sound and carious dentin in the control group and after radiopaque tagging (n = 12/group). Different letters indicate statistical significance between groups (p < 0.001). Box and line: 25th/75th percentiles and median, whiskers: minimum and maximum.

Micro-tensile bond strength was significantly lower on carious than sound dentin (p < 0.01) regardless of the treatment group (Fig. 5). Tagging led to significantly (p < 0.01) decreased bond strengths on sound dentin (median differences were −22% and −33% for SnCl_2_ and SnF_2_, respectively). This was confirmed for carious dentin as well (−50% and −54% reductions for SnCl_2_ and SnF_2_, respectively). When assessed for fracture mode, all samples showed adhesive fractures between adhesive and dentin.

### Scanning electron microscopy and EDS

**Figure 6:**
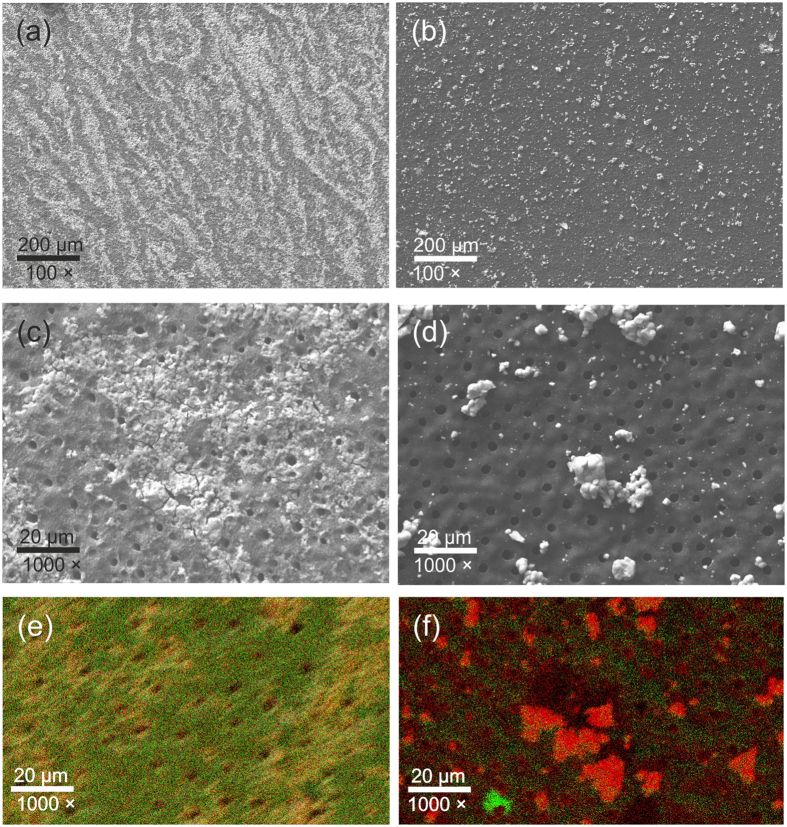
Scanning electron microscopic and EDS images of etched dentin tagged with stannous chloride (**a,c,e**) and stannous fluoride (b,d,f). Stannous chloride precipitations covered the dentin relatively homogenously as can be seen at 100× (**a**) and 1000× (**b**) magnification. EDS analysis confirmed both tin (in red/orange) and chloride (green) to be dispersed. Stannous fluoride precipitations were more localized, as can be seen in (**b,d**). However, when assessed via EDS (f: red: tin, green: fluoride), both tin and fluoride were seen in detectable concentrations all over the dentin.

Scanning electron microscopy found the dentin to be homogeneously covered by stannous chloride (Fig. 6). This was confirmed via EDS. In contrast, stannous fluoride precipitations seemed more localized when assessed microscopically, but were found in detectable concentrations all over the dentin as well using EDS (Fig. 6).

## Discussion

Radiopaque tagging might allow to reduce the diagnostic uncertainty associated with selective excavation and thereby increase the acceptance of this treatment strategy among dentists. However, prior to clinical application, a number of questions need to be answered. These pertain to the applicability of the technique as well as its benefits (for example with regards to antibacterial effects) and safety (dentin bond strength, toxicity, pulpal reaction). This study assessed if tagging effects depended on the depths of the carious lesions, and investigated if tagging exerts antibacterial effects on sealed lesions or affects dentin bond strengths of conventional etch-and-rinse adhesives.

We found that relative tagging effects decreased with increasing lesion depth. We assume the transport of the tagging fluid (and the containing tin ions) to be diffusion driven. It seems possible that the application time (which would need to be short in a clinical setting) did not allow for full penetration of lesions (which extended up to 700 μm deep). Moreover, surface precipitations of tin salts (as discussed later on) could lead to decreasing tin concentrations in deeper lesion areas and could impede ion transport into the lesion depth. This would mean decreased effectiveness of tagging if large amounts of (rather soft) dentin remained after carious tissue removal. This could limit the indication of tagging, as especially deep lesions are well detectable radiographically and in special need of tagging. The used method to assess tagging effects has a number of limitations: First and foremost, induced lesions were aligned nearly perfectly perpendicular to the radiographic beam. That allowed to precisely measure tagging effects, but would not necessarily be the case *in vivo*. Instead, clinically, a three-dimensional overlap of tagged areas on two-dimensional radiographs is likely, which might help to explain why tagging of natural deep lesions seems more successful than tagging of deep lesions in the present study^6^. Second, lesions were artificially induced. While the used protocol was shown to induce lesions with similar mineral loss profile as natural residual lesion^12^, it can be assumed that natural lesions differ for example in their surface behavior (like the presence of a smear layer) as well as the integrity of organic components. Both the degree of demineralization and the degradation of organic collagen components have been shown to impact on penetration of tin solutions into the deeper areas of the dentin^8^. Moreover, while we prevented desiccation of dentin samples during application, no pulpal fluid simulation was performed (as this was not feasible using T-WIM samples). Clinically, fluid extrusion and the inherent moistness of dentin could decrease tagging effects even further (although full tubular integrity and continuity, both partially being a prerequisite for fluid pressure effects, are unlikely in natural lesions). It should be noted that previous experiments showed successful tagging even under fluid pressure application^6^. Overall, it will be required to validate our findings on natural lesions *in vitro* (a clinical validation using repeated radiographic assessment will not be justifiable ethically). Moreover, an optimization of the application protocol might be required. For example, pretreatment of the lesion could be performed, reducing moisture and increasing permeability. Tagging materials could be dissolved in a different medium, for example ethanol, which could allow deeper transport of tin-containing fluids into the lesion before binding to collagen and immobilization. Last, restorative materials releasing radiopaque materials could be developed, leading to decreasing radioluency of sealed lesion with time.

Such restorative materials would most likely have antibacterial properties as well. We found radiopaque tagging to reduce viable bacteria numbers within artificially bacterially-loaded dentin lesions below the detection threshold of cultivation. More important, this reduction was significantly higher than that of CHX, which is often seen as standard for cavity disinfection. Similar as for CHX, cavity pretreatment using tin compounds might also inhibit matrix metallo-proteinases^13^, which could have additional benefits with regards to stability of the dentin-adhesive interface, reducing leakage and increasing the longevity of restorations^14^,^15^,^16^. We used an invasion model which builds on pre-demineralization to allow faster bacterial invasion, with the resulting lesions being extremely deep and demineralized. However, bacterial loads within dentin were similar to those found in sealed lesions *in vivo*^17^,^18^. The external validity of the model is nevertheless limited, as effects on only one cariogenic strain were measured. In this sense, caution is required when deeming CHX unsuitable for cavity disinfection, as its effects on *L. rhamnosus* might not be transferable to other species in complex biofilms^19^,^20^,^21^. Given that we did not find any viable bacteria to remain after applying radiopaque tagging, it is unlikely that other antibacterial substances would exceed the bactericidal effects of the used tagging materials. Notable, the clinical relevance of any such cavity disinfection remains unclear given the effects of sealing itself on bacterial survival^22^. Moreover, application of phosphoric acid during conditioning or acidic primers during bonding procedures could have an antibacterial effect, too, and our findings should be put into perspective accordingly by future studies.

Our study found dentin bond strengths of one (gold standard) etch-and-rinse adhesive (OptiBond FL) to be significantly reduced in tagged compared with non-tagged sound and carious surfaces. We tested bonding to both substrates, as clinically sound would dentin could be found in the periphery, while demineralized or infected and degraded dentin would be found after selective excavation^23^. Using microscopic and EDS analysis, we found a tin impregnation of the dentin to possibly decrease penetration of the resin primer into the collagen network, thereby weakening bond strengths. Notably, for SnCl_2_, we found this material to be widely dispersed, with microscopic analyses showing streaks of the material reflecting its application mode. In contrast, SnF_2_, was found in more localized precipitations, which we ascribe to this material being significantly lower concentrated. However, using EDS we could show that even here, tin and fluoride ions were found in large dentin areas, which explains why bond strength decreases where only limitedly different between materials. Such precipitations, consisting for example Sn_2_(PO_4_)OH, Sn_3_F_3_PO_4_, or Ca(SnF_3_)_2_^24^,^25^,^26^, have been found before when using stannous chloride solutions^7^,^8^. The inhibition resulting from applying this solution has also been attributed to precipitations inhibiting primer diffusion^7^. Interestingly, the opposite (increased bond strengths) has been found when low concentrations of stannous chloride were applied prior to bonding using 10-methacryloyloxydeycl dihydrogen phosphate (MDP) containing adhesives. This has been attributed to tin possibly promoting “docking” of MDP to collagen^7^,^24^, or tin occupying negatively charged bonding sites in demineralized collagen, thereby decreasing the polarity of the organic matrix and increasing its accessibility for adhesives^24^. It remains unclear if similar effects would be found after applying high concentrated tin solutions. We had used Optibond FL, a non-MDP containing adhesive, as it regarded as gold standard, but also as we aimed to describe the possible “worst case” of bond strength decrease. Based on our and the aforementioned findings, it could be expected that such bond strength decreases are less pronounced when using MDP-containing adhesives. As a result of our findings, an application protocol needs be developed which allows to circumvent the observed dentin bond strength reductions. Mere rinsing of the surface will not be able to remove the precipitations^6^. As these precipitations are acid-soluble, etching after instead of before tagging might be a solution^6^,^8^, but could decrease penetration and thereby hamper the tagging effect. Decreasing the concentration will only be limitedly possible given the resulting decreased tagging effect^6^. A different solvent, which could allow to better remove any ion-containing fluids from the surface before any wide precipitation has begun, might be an option; we discussed ethanol as one means to achieve this.

In conclusion, radiopaque tagging might help to overcome the diagnostic uncertainty associated with residual lesions as a result of selective excavation. This would in turn help to increase acceptability of this treatment strategy. While we found tagging to exert beneficial antibacterial effects, we also demonstrate the limitations of tagging with regards to both its masking effects in deeper lesions as well as the resulting bond strength decreased of tagged compared with untagged sound or carious dentin. An optimized application protocol needs to be developed and further investigations into the biologic safety of tagging are required before clinical application can be recommended.

## Additional Information

**How to cite this article**: Umwali, A. *et al.* Radiographic, antibacterial and bond-strength effects of radiopaque caries tagging. *Sci. Rep.* **6**, 27319; doi: 10.1038/srep27319 (2016).
